# Blood Harmane Concentrations in 497 Individuals Relative to Coffee, Cigarettes, and Food Consumption on the Morning of Testing

**DOI:** 10.1155/2011/628151

**Published:** 2011-05-16

**Authors:** Elan D. Louis, Pam Factor-Litvak, Marina Gerbin, Wendy Jiang, Wei Zheng

**Affiliations:** ^1^GH Sergievsky Center, College of Physicians and Surgeons, Columbia University, Unit 198, Neurological Institute, 710 West 168th Street, New York, NY 10032-2699, USA; ^2^Department of Neurology, College of Physicians and Surgeons, Columbia University, New York, NY 10027-6900, USA; ^3^Department of Epidemiology, Mailman School of Public Health, Columbia University, New York, NY 10032-3727, USA; ^4^Taub Institute for Research on Alzheimer's Disease and the Aging Brain, College of Physicians and Surgeons, Columbia University, New York, NY 10032, USA; ^5^School of Health Sciences, Purdue University, West Lafayette, IN 47907-2051, USA

## Abstract

Harmane, a potent neurotoxin linked with several neurological disorders, is present in many foods, coffee, and cigarettes. We assessed whether morning food/coffee consumption and smoking were reflected in blood harmane concentrations (BHCs) we obtained in an epidemiologic sample (*n* = 497). Participants who smoked on the morning of phlebotomy had similar logBHCs to those who had not smoked (*P* = .57); there was no correlation between logBHCs and number of cigarettes (*P* = .59). Among the coffee drinkers, there was no correlation between number of cups and logBHCs (*P* = .98). Participants who had eaten on the morning of phlebotomy had similar logBHCs to those who had not (*P* = .49); logBHCs did not correlate with the time latency between last food consumption and phlebotomy (*P* = .74). BHCs in this sample of ~500 individuals did not covary with recent smoking, coffee, or food consumption, suggesting that our inability to withhold these exposures on the morning of phlebotomy was not reflected in the BHCs we measured.

## 1. Introduction

Harmane (1-methyl-9H-pyrido[3,4-*β*]indole) is a potent neurotoxin that has been linked with several neurological outcomes [1, 2]. Although it is produced endogenously by the body, harmane is also present in many foods (esp. meats but also plant-derived foods) [3]. Studies have shown that harmane concentrations are particularly high in certain commonly consumed beverages (esp. coffee) [3–6] as well as cigarettes [3, 5, 7]. Coffee consumption and smoking are widespread and common human behaviors. 

 Smoking [8] and food ingestion [9] have been shown to result in transient elevations in BHCs. After smoking, blood harmane levels rise rapidly and seem to return to baseline within one hour, although the number of tested human volunteers has been small (*n* = 3) [8]. After oral dosing (harmane dissolved in corn oil), blood harmane levels in rats peaked rapidly (in approximately 30 minutes) and then gradually returned to baseline within 3–5 hours [9]. 

 Studies of harmane and its relation to neurological outcomes (essential tremor [ET] and Parkinson's disease) often involve work with frail and elderly study subjects for whom fasting on the morning of testing is not feasible, especially as many of these patients must also take prescription medications (often accompanied with food). It is also difficult to ask smokers to refrain, and their attempts to do so can transiently exacerbate their tremor, confounding the accurate assessment of tremor severity. Given these limitations, it is important to know whether food consumption, coffee consumption, and/or smoking on the morning of phlebotomy are reflected in blood harmane concentrations (BHCs).

 Our overarching question was whether morning food/coffee consumption and smoking were reflected in the BHCs we obtained. The specific questions we asked were the following. (1) Did participants who smoked on the morning of phlebotomy have higher BHCs than those who did not smoke? (2) Was the the number of cigarettes smoked on the morning of phlebotomy correlated with BHCs? (3) Did participants who consumed coffee on the morning of phlebotomy have higher BHCs than those who did not? (4) Was the number of cups of coffee on the morning of phlebotomy correlated with BHCs? (5) Was there a correlation between the time of last food ingestion and BHCs? If these questions were answered affirmatively, this would suggest that these exposures are important to consider when assessing case-control differences in BHCs. If not, it would suggest that these exposures are relatively unimportant in this context. Using data from a large epidemiological study of ET, we evaluated these exposures in approximately 500 individuals. In addition to BHCs, information on smoking, coffee consumption, and food intake on the morning of phlebotomy were available.

## 2. Methods

### 2.1. Participants

All participants were enrolled between June 2000 and May 2008 in a study of the environmental epidemiology of tremor at Columbia-University Medical Center (CUMC). Participants consisted of ET cases and controls. By design, ET cases were identified from several sources; the major ones were a computerized billing database of patients at the Neurological Institute of New York, CUMC, and the International Essential Tremor Foundation, whose members were mailed advertisements [2, 10]. All cases had received a diagnosis of ET from their treating neurologist and lived within two-hour driving distance of CUMC in the New York Metropolitan area. Based on a videotaped tremor examination, described below, their diagnoses were confirmed by a senior movement disorder neurologist (E.D.L.) using published diagnostic criteria (moderate or greater amplitude action tremor during ≥3 activities or a head tremor in the absence of Parkinson's disease, dystonia, or another neurological disorder) [10–12]. 

 Normal control subjects were also recruited during the same time period [2, 10]. These controls were identified using random digit telephone dialing within a defined set of telephone area codes in the New York Metropolitan area that were represented by the ET cases. Controls were frequency matched to cases based on gender, race, and current age. 

 The CUMC Internal Review Board approved of all study procedures, and signed written informed consent was obtained from all participants upon enrollment [2, 10].

 Of 698 ET cases and controls enrolled, complete data were available in 497 (71.2%). The majority of the remaining participants had refused phlebotomy or had had an unsuccessful phlebotomy attempt. The final sample of 497 was similar to the base sample of 698 in terms of age (mean ± standard deviation = 66.0 ± 14.4 versus 67.2 ± 14.2 years, *t* = 1.43, and *P* = .15), gender (267 [53.7%] versus 376 [53.9%] female, chi-square = 0.00, and *P* = .96), years of education (15.4 ± 3.5 versus 15.3 ± 3.5 years, *t* = 0.49, and *P* = .63), and proportion who were current smokers (43 [8.7%] versus 57 [8.2%]) chi-square = 0.09, and *P* = .77).

### 2.2. Clinical Evaluation

All participants were evaluated in person by a trained tester. The tester administered clinical questionnaires and performed a videotaped tremor examination and phlebotomy. 

 As noted above, most evaluations were home visits and, therefore, were performed in the late morning, making fasting BHCs impractical. 

 The tester used a structured questionnaire to collect demographic information including age in years, gender, race, years of education, current smoker (yes versus no), the number of cigarettes smoked per day, cigarette pack years, the number of cigarettes smoked on the morning of phlebotomy, and the number of cups of coffee consumed on the morning of phlebotomy. Several years into the study, questions were added as to whether food was consumed on the morning of phlebotomy and the number of hours between last food consumption and the phlebotomy. Similar data on number of hours between smoking or coffee consumption and phlebotomy were not available. Medical comorbidity was assessed with the cumulative illness rating scale (range = 0–42 (high comorbidity)) [13].

 The tester videotaped a tremor examination in all participants [10, 12]. Each of 12 videotaped action tremor items was rated by a senior movement disorder neurologist (E.D.L.) on a scale from 0 (none) to 3 (severe tremor) [10–12].

### 2.3. BHCs

At the time of the evaluation, phlebotomy was performed. When the evaluation was performed in the participant's home, blood samples were temporarily stored on ice packs and then several hours later transferred to a −20°C freezer; if performed at CUMC, they were placed immediately into a −20°C freezer. Blood harmane concentrations were measured blinded to all clinical information with a well-established high-performance liquid chromatography method described in detail in our previous studies [2, 10, 14]. The intraday precision, measured as a coefficient of variation at 25 ng/mL, was 6.7%. The interday precision was 7.3% [14]. Our method uses whole blood rather than plasma. Harmane is highly lipophilic, accumulating inside of blood cells, with published studies demonstrating low relative recovery of harmane from plasma [9, 14].

### 2.4. Statistical Analyses

Statistical analyses were performed in SPSS (Version 18.0). The empirical distribution of BHCs was positively skewed (one-sample Kolmogorov-Smirnov test, *z* = 9.37, *P* < .001), even after log transformation (one-sample Kolmogorov-Smirnov test, *z* = 2.37, *P* < .001). Hence, nonparametric tests (Mann Whitney U, Kruskal-Wallis, Spearman's rho) were used when assessing this variable. For smoking, pack years was assigned the value “0” for nonsmokers. We stratified participants based on tertiles of cigarettes smoked on the morning of phlebotomy. For coffee consumption, we dichotomized participants based on highest consumption level (≥4 cups per day).

To assess potential confounding variables (e.g., education), stratified analyses were performed.

## 3. Results

### 3.1. Introduction

The 497 participants (242 ET cases and 255 controls) had a mean age of 66.0 ± 14.4 years (Table 1). Of the 497, 43 (8.7%) had smoked on the morning of phlebotomy (0.4 ± 1.5, range = 0–13 cigarettes), and 214 (43.1%) had consumed coffee on the morning of phlebotomy (0.7 ± 1.0, range = 0–5 cups). The mean log BHCs was 0.51 ± 0.56 g^−10^/mL in ET cases and 0.38 ± 0.63 g^−10^/mL in controls, a difference that was statistically significant (Mann Whitney *P* = .01). The non-log-transformed BHCs was 11.49 ± 52.05 (median = 2.49)  g^−10^/mL in ET cases and 10.42 ± 56.16 (median = 1.81) g^−10^/mL in controls. Log BHCs were not associated with age (Spearman's *r* = 0.02, *P* = .68), gender (Mann Whitney *P* = .80), white race (Mann Whitney *P* = .96), or cumulative illness rating scale score (Spearman's *r* = −0.001, *P* = .99), but they were weakly and inversely associated with years of education (Spearman's *r* = −0.09, *P* = .04).

### 3.2. Smoking

Current smokers did not differ from current nonsmokers in terms of log BHCs (0.39 ± 0.52 [median = 0.20] versus 0.44 ± 0.62 [median = 0.34], Mann Whitney *z* = 0.64, *P* = .52). Additionally, neither cigarette pack years (Spearman's *r* = −0.04, *P* = .42) nor number of cigarettes smoked per day (Spearman's *r* = 0.04, *P* = .82) were correlated with log BHCs. 

 Participants who smoked on the morning of phlebotomy had similar log BHCs to those who had not smoked on that morning (0.41 ± 0.51 [median = 0.30] versus 0.45 ± 0.61 [median = 0.34], Mann Whitney *z* = 0.58, *P* = .57). There was no correlation between log BHCs and the number of cigarettes smoked on the morning of phlebotomy (Spearman's *r* = −0.03, *P* = .59), even after restricting the analyses to the 43 participants who smoked on the morning of phlebotomy (Spearman's *r* = 0.10, *P* = .54). High smokers (i.e., the 15 participants who were in the upper tertile of cigarettes smoked on the morning of phlebotomy, i.e., ≥5 cigarettes) had similar log BHCs to the participants who had not smoked on the morning of phlebotomy (0.51 ± 0.54 [median = 0.20] versus 0.45 ± 0.61 [median = 0.34], Mann Whitney *z* = 0.20, *P* = .84). 

 Years of education, which were associated with log BHCs, were inversely associated with number of cigarettes smoked on the morning of phlebotomy (Spearman's *r* = −0.12, *P* = .008). Therefore, participants were stratified based on median years of education into a high (≥16 years) versus low (<16 years) education group; within these education strata, there was no difference in the log BHCs between participants who had versus who had not smoked on the morning of phlebotomy (for high education stratum, Mann Whitney z = 0.92, *P* = .36; for low education stratum, Mann Whitney *z* = 0.05, *P* = .96). Participants were also stratified into those who had versus those who had not consumed coffee on the morning of phlebotomy; within these strata, there was no difference in the log BHCs between participants who had versus who had not smoked on the morning of phlebotomy (for coffee drinkers, Mann Whitney *z* = 0.31, *P* = .76; for nondrinkers, Mann Whitney *z* = 0.90, *P* = .37). Finally, participants were stratified into cases versus controls; within these strata, there was no difference in the log BHCs between participants who had versus who had not smoked on the morning of phlebotomy (for cases, Mann Whitney *z* = 1.21, *P* = .23; for controls, Mann Whitney *z* = 0.30, *P* = .76).

### 3.3. Coffee

Participants who drank coffee on the morning of phlebotomy had a marginally lower log BHCs than those who had not had coffee on that morning (0.39 ± 0.59 [median = 0.29] versus 0.49 ± 0.61 [median = 0.38], Mann Whitney *z* = 1.82, *P* = .069). ET cases had higher blood harmane than controls and, presumably to avoid exacerbating their tremor, also drank less coffee than controls. After stratifying by case-control status, drinking coffee on the morning of phlebotomy did not seem to be associated with higher log BHCs (Table 2). 

 In the combined sample of cases and controls, there was a marginal inverse correlation between log BHCs and the number of cups of coffee consumed on the morning of phlebotomy (Spearman's *r* = −0.08, *P* = .08) but not when analyses were restricted to the 214 participants who had consumed coffee on the morning of phlebotomy, Spearman's *r* = 0.002, *P* = .98. High coffee consumers (i.e., the 11 participants who drank ≥4 cups of coffee on the morning of phlebotomy) had marginally lower log BHCs than participants who had not had coffee on the morning of phlebotomy (0.38 ± 0.66 [median = 0.29] versus 0.49 ± 0.61 [median = 0.38], Mann Whitney *z* = 1.72, *P* = .085). After stratifying by case-control status, there was a marginal, weak, inverse association between the number of cups of coffee and log BHCs in cases only but not when the analyses were restricted to the participants who had had coffee on the morning of phlebotomy (Table 2). 

 Years of education, which were associated with log BHCs, were not associated with the number of cups of coffee consumed on the morning of phlebotomy (Spearman's *r* = 0.008, *P* = .86).

### 3.4. Food Consumption

Data were available on food consumption on the morning of phlebotomy in 215 participants, of whom 196 (91.2%) had eaten on the morning of phlebotomy. The 196 participants who had eaten on the morning of phlebotomy had similar log BHCs to the 19 who had not eaten (0.35 ± 0.44 [median = 0.33] versus 0.34 ± 0.47 [median = 0.24], Mann Whitney *z* = 0.70, and *P* = .49). After stratifying by case-control status, the results were similar in cases: (0.42 ± 0.47 [median = 0.38] in 115 eaters versus 0.37 ± 0.47 [median = 0.24] in 7 noneaters, Mann Whitney *z* = 0.48, and *P* = .63) and in controls (0.25 ± 0.39 [median = 0.26] in 81 eaters versus 0.32 ± 0.48 [median = 0.24] in 12 noneaters, Mann Whitney *z* = 0.18, and *P* = .86).

 Data were available for 109 participants on the time lapse between last food consumption and phlebotomy. The mean lapse was 2.0 ± 1.1 hours [median = 1.8], range = 0–5.5 hours. There was no correlation between the duration of this time lapse and log BHCs (Spearman's *r* = 0.03, *P* = .74). After stratifying by case-control status, the results were similar (Spearman's *r* = 0.13, *P* = .36 among 51 controls; Spearman's *r* = −0.02, *P* = .91 among 58 cases).

**Figure 1:**
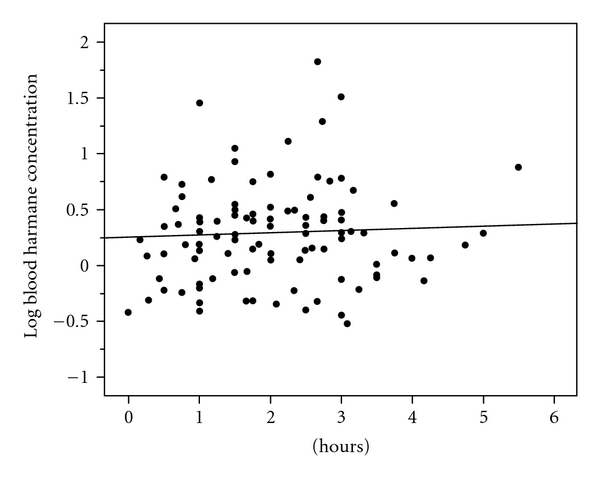
Hours between food consumption and phlebotomy (*x* axis) and log blood harmane concentration (g^−10^/ml) (*y* axis). Regression line is shown (Spearman's *r* = 0.03, *P* = .74).

 In the 109 participants, we stratified the time lapse into hourly categories, and log blood harmane was similar in participants who had eaten one hour or less prior to phlebotomy and participants who had eaten 2, 3, 4, 5, or more hours prior to phlebotomy (Kruskal-Wallis *P* = .19, Table 3, Figure 1). 

## 4. Discussion

Harmane is a beta-carboline alkaloid that has been linked with several neurological diseases [1, 2], making this potent neurotoxin of etiological interest in these diseases. Yet, studies of harmane and its relation to neurological diseases such as ET and Parkinson's disease generally require work with elderly subjects who are fragile or in poor health and for whom fasting blood levels are either impractical or simply not possible. Furthermore, withholding cigarettes can increase anxiety and discomfort, thereby exacerbating tremor and confounding the accurate assessment of tremor severity. These practical limitations lead to the research question whether food consumption, coffee consumption, and/or smoking, which often occur on the morning of phlebotomy, influence BHCs. The current dataset allowed us to examine this set of questions in a sample of approximately 500 study participants with both exposure data on these lifestyle characteristics and data on BHCs. 

 We found that participants who had smoked on the morning of phlebotomy had similar BHCs to their counterparts who had not smoked that morning. Furthermore, there was no detectable association between dosage of exposure to cigarettes on the morning of phlebotomy and BHCs. A cautionary note is that we did not collect blood samples within one hour of smoking, and, as discussed in detail below, this could have accounted for the lack of association between smoking and BHCs. For coffee consumption, there was only a very marginal inverse relationship with BHCs, but this was not consistent and, hence, of questionable significance. Finally, in the subsample of participants with timed data on food consumption, there was no discernable correlation between the time of the last food ingestion and BHCs. These results were observed in parallel in normal controls as well as in a group of ET patients. 

 How do our results relate to those from prior studies? After smoking, blood harmane levels rise rapidly and seem to return to baseline within one hour [8], which suggests that the rise is transient and time limited. In another study of 39 human volunteers, plasma concentrations of norharman (9H-pyrido[3,4-*β*]indole), a neurotoxin that is structurally very similar to harmane, were on average several fold higher when smokers were not restricted in terms of smoking as compared to a condition in which the same smokers had refrained from smoking for at least six hours [15]. Another study [16], of 20 human volunteers, found a positive correlation between number of cigarettes smoked per day and plasma harmane concentration. It is possible that participants in these two prior studies smoked with one hour of phlebotomy, thereby resulting in acute elevations in BHCs and a detectable correlation between smoking behavior and BHCs. We did not collect data on the time that our participants smoked, so we do not know how many smokers smoked within one hour of phlebotomy. If most did not, this could explain our null finding. 

 In rats, oral dosing of harmane (i.e., 110 umol/kg harmane dissolved in corn oil) caused blood harmane levels to peak rapidly (in approximately 30 minutes) and then gradually return to baseline within 3–5 hours [9]. Yet, a study involving humans showed a range of responses to ingestion of ethanol or orange juice, including transient declines and, in others, transient rises in BHCs, with these transient changes ranging in duration from 1 hour to more than 8 hours [17]. Hence, the effects of food ingestion on BHCs in humans are less clear than the effects of oral dosing of harmane in laboratory animals. Our current results did not find any association between food intake or time of food intake and BHCs.

 This study had limitations. This was not a pharmacokinetic study, and we did not assess pre- versus post-consumption BHCs in order to directly test whether blood concentrations rose. Furthermore, while in a subsample of participants, we had data on the time latency between the last food ingestion and phlebotomy, we had no data on the time of the last cigarette use or coffee consumption and phlebotomy, so for cigarettes and coffee, we were unable to time lock the events to phlebotomy. Second, we limited our assessment of smoking to cigarettes (i.e., inhaled smoke products) rather than pipe smoke or chewing tobacco, which were rare. Third, we did not assess whether different brands of coffee or cigarettes or differences in cooking styles influenced the BHCs we observed. The study also had several strengths. First, both BHCs and clinical data were assessed in the same study sample, comprising a collection of nearly 500 individuals. Second, for smoking, we presented both historical data for the degree of their regular consumption as well as data on acute consumption on the morning of phlebotomy. Third, the results were observed in normal controls and then reproduced in ET patients. Fourth, most published studies determined harmane concentrations in plasma, whereas our study investigates harmane in whole blood. The latter approach allows for a more accurate assessment of harmane concentrations by including all blood compartments. Finally, there is the uniqueness of the question; there are no other studies that have examined this issue.

## 5. Conclusions

In summary, BHCs in this sample of nearly 500 individuals did not covary with smoking, coffee consumption, or food consumption, suggesting that our inability to withhold these exposures on the morning of phlebotomy was not reflected in the BHCs we measured.

##  Financial Disclosure

E. D. Louis was funded by R01 NS39422, P30 ES09089, and RR00645 (General Clinical Research Center) from the National Institutes of Health (Bethesda, MD and Research Triangle, NC). P. Factor-Litvak was funded by R01 ES12231 and R01 ES017024 from the National Institutes of Health (Research Triangle, NC). W. Zheng was funded by R01 NS39422, R01 ES008146, and R21 ES017055 from the National Institutes of Health (Research Triangle, NC). The National Institutes of Health played no role in the study design, the collection of data, the analysis and interpretation of data, the writing of the paper, or in the decision to submit the paper for publication. The authors were free to design, conduct, interpret, and publish research, and this was not compromised by the National Institutes of Health.

##  Conflict of Interests 

The authors declare that there is no conflict of interests. The statistical analyses were conducted by E. D. Louis.

## Figures and Tables

**Table 1:** Characteristics of 497 study participants.

Age (years)	66.0 ± 14.4
Female gender	267 (53.7)
White race	445 (89.5)
Education (years)	15.4 ± 3.5
Cumulative illness rating scale score	5.3 ± 3.7
Current smoker	43 (8.7)
Number of cigarettes smoked per day (smokers only)	18.8 ± 17.0
Cigarette pack years	10.8 ± 20.9
Smoked on morning of phlebotomy	43 (8.7)
Number of cigarettes smoked on morning of phlebotomy	0.4 ± 1.5
Drank coffee on morning of phlebotomy	214 (43.1)
Number of cups of coffee on morning of phlebotomy	0.7 ± 1.0
Log blood harmane concentration in g^−10^/ml	0.45 ± 0.60

Values are means ± standard deviation or number (percent).

**Table 2:** Log blood harmane concentration and coffee consumption in analyses stratified by case-control status.

	ET cases (*N* = 242)	Controls (*N* = 255)
Log blood harmane concentration (g^−10^/ml)		
Drank coffee on morning of phlebotomy	0.42 ± 0.54 [0.34]	0.37 ± 0.62 [0.28]
Did not drink coffee	0.56 ± 0.57 [0.42]	0.40 ± 0.65 [0.26]
	*P* = .11^a^	*P* = .69^a^

Correlation between the number of cups of coffee on morning of phlebotomy and log blood harmane concentration	*r* = −0.012, *P* = .068	*r* = −0.002, *P* = .97

Correlation between the number of cups of coffee on morning of phlebotomy and log blood harmane concentration (in 214 participants who drank coffee on the morning of phlebotomy)	*r* = −0.014, *P* = .23	*r* = 0.078, *P* = .38

^a^Mann Whitney test. In upper row, values are means ± standard deviation (median). Correlation coefficients, *r*, are Spearman's rho.

**Table 3:** Log blood harmane concentrations by categories of time lapse (hours) between food consumption and phlebotomy.

Time lapse (hours) between food consumption and phlebotomy	*N*	Log blood harmane concentration (g^−10^/ml)
≤1	31	0.20 ± 0.42 [0.19]
>1 to 2	30	0.33 ± 0.34 [0.38]
>2 to 3	31	0.40 ± 0.53 [0.40]
>3 to 4	12	0.10 ± 0.33 [0.06]
>4	5	0.26 ± 0.38 [0.19]

Values are means ± standard deviation (median).

## Abbreviations

BHCs: Blood harmane concentrations
CUMC: Columbia-University Medical Center
ET: Essential tremor.
